# Pills and the damage done: the opioid epidemic as man-made crisis

**DOI:** 10.3389/fpubh.2023.1241404

**Published:** 2024-01-12

**Authors:** Rebecca McDonald, Desiree Eide, Svetlana Skurtveit, Thomas Clausen

**Affiliations:** ^1^Norwegian Centre for Addiction Research (SERAF), Institute of Clinical Medicine, University of Oslo, Oslo, Norway; ^2^Department of Chronic Diseases, Norwegian Institute of Public Health, Oslo, Norway

**Keywords:** analgesics, overdose, misuse, prescription opioids, poisoning, oxycodone, addiction

## Abstract

The prescription opioid epidemic has slowly evolved over the past quarter century with increasingly detrimental consequences for public health. Man-made crises are often unforeseen and characterized by a situation without natural causes where – because of human intent, error, negligence, or the failure of manmade systems – the level of needs in the population exceeds available resources to counter the problem. This paper presents the prescription opioid epidemic as a man-made crisis and explores the public health impact of opioid manufacturers and other industries producing commodities with addictive potential as a shared vulnerability among countries. We examine this concept within the framework of the commercial determinants of health. We address three key aspects of the commercial determinants of health: (1) Cross-industry mechanisms, (2) policy inertia, and (3) the role of industry in science. Within cross-industry mechanisms, we explore parallels between prescription opioid epidemic and unhealthy commodity industries in terms of marketing, corporate use of misinformation, and diversionary tactics. Next, we examine how policy inertia has dominated the slow response to this man-made crisis. Lastly, we discuss how results from clinical trials are used as a key marketing strategy for drugs. The origins of the prescription opioid epidemic may be traced to innovations in drug development with the promise of improved pain management. However, through multiple factors, including fraudulent marketing from pharmaceutical industry and policy inertia, the resulting crisis represents a multi-system failure of regulation exploited by corporate greed.

## 1 Introduction

In this “Perspective” piece, we present the prescription opioid epidemic as man-made crisis within the framework of the commercial determinants of health (CDOH). By definition, man-made crises lack natural causes and result from human intent, error, negligence, or the failure of manmade systems (1). Prescribers are recognized to have played a central role in generating the oversupply of opioids (2). However, we cannot look at prescriber behavior in isolation, without considering how pharmaceutical industry benefitted from weak regulations and influenced prescriber education to advance its agenda of opioid sales, at the expense of the lives of thousands (3). The impact of corporate action on health outcomes is indeed focus of the CDOH, a concept first proposed by West and Marteau in 2013 (4) as ‘factors that influence health which stem from the profit motive’. At the example of the tobacco industry and cigarette sales, West and Marteau describe the corporate profit motive as detrimentally opposed to public health.

A wide range of definitions of the CDOH now exists, which a recent consensus paper in The Lancet (5) integrates as: “the systems, practices, and pathways through which commercial actors drive health and equity.” This neutral definition acknowledges that prescription opioids can have both positive and negative effects on human health. Indeed, Babor and Ferreira-Borges describe the opioid epidemic as a “prime example of the growing importance of CDOH” (3).

In the United States (US), the epicenter of the epidemic, prescription opioid overdose deaths have quadrupled since 1999 and been directly linked to the country’s declining life expectancy (6). The resulting level of treatment needs for opioid use disorder exceeds available resources to counter the problem (7). Looking beyond North America, misuse of opioid analgesics is now spreading globally and increasingly affecting parts of the Middle East, Africa, and Northern Europe, including Scandinavia (8, 9). As a team of authors, we are based in Norway, where prescription opioids have overtaken heroin as the most frequent cause of overdose deaths since 2016 (10, 11). Despite these trends, the onset of an opioid epidemic of North American proportions currently appears unlikely (12, 13). The reason might be found in differences in the European system (e.g., access to free or low-cost healthcare, fewer automatic prescription refills, lack of direct-to-consumer advertising for prescription medications) (14, 15).

Our aim here is not to appraise such differences, but to use the CDOH framework to describe the potential influence of the opioid industry (and other industries producing commodities with addictive potential) on public health as a shared vulnerability among countries. The core subject is to improve the understanding of how the corporate profit motive has driven opioid overprescribing and mortality. Thus, we apply three key aspects of the CDOH to the opioid epidemic: (1) Cross-industry mechanisms in the marketing of potentially addictive products, (2) policy inertia and lack of government intervention as evidence of health harm emerges, and (3) the role of industry in science.

### 1.1 Cross-industry mechanisms: what parallels does the prescription opioid epidemic have with other industries that create significant health damage?

#### 1.1.1 Parallels in marketing with unhealthy commodity industries

Alcohol, tobacco, and ultra-processed foods (UPF) are typically considered unhealthy commodities, i.e., products that *per se* cause significant health damage, “aimed at, and accessible to, large numbers of consumers,” and “highly profitable because of their low production cost, long shelf-life, and high retail value” (16, 17).

Due to their intended medical use, prescription opioids do not constitute unhealthy commodities. Their prescription-only status also makes pharmaceutical opioids less accessible than alcohol and tobacco (legal access restrictions by age and/or retail venue) and UPF (no restrictions), thus attracting a much smaller customer base. Nonetheless, several cross-industry similarities in marketing can be identified.

Firstly, alcohol, tobacco, and prescription opioids are all commodities with potential for addiction-driven consumption (18). Consumers addicted to these commodities tend to consume at least daily and in greater amounts than non-addicted consumers, meaning that their excess consumption drives consumer spending and accounts for most corporate profits (“addiction surplus”) (18). As Adams and Livingstone illustrate (19), corporations are invested in establishing daily use early in customers’ lives and maintaining individual excess consumption. To this end, corporations use misinformation to lobby against changes in legislation or clinical guidelines that seek to limit product access (19).

Secondly, due to their low production cost, low mass (135 mg), and no storage requirements (20, 21), OxyContin (and other prescription opioids) are ideal for international export. Indeed, in the 2010s, as US sales of OxyContin by the Sackler family-owned Purdue Pharma were stagnating because of prescribing restrictions and possible saturation of the domestic market (22), opioid industry began to target low- and middle income countries (LMIC). A 2016 LA Times investigation (23) reported that Mundipharma, i.e., a conglomerate of companies also belonging to the Sacklers, has undertaken lobbyism activities “right out of the playbook of Big Tobacco” to expand OxyContin into markets in Africa, Asia, Latin America, and the Middle East. In addition to hosting seminars targeting prescribers, this has involved aggressive marketing such as patient discounts for prescription opioids (foot-in-the-door technique) as well as policy interference in the context of poor legislation and regulation in LMICs, driving opioid consumption in places ill-prepared to deal with its negative impacts on public health (23). The provision of product discounts can have devastating unintended consequences in LMIC. In the 1970s, thousands of infants died from malnutrition after Nestlé had distributed free product samples of infant formula to parents in African and Asian hospitals (24). Mothers ceased breastfeeding (considering it inferior to formula), mixed formula with unclean water, or diluted it too much after the free samples had run out (24).

#### 1.1.2 Corporate use of misinformation

In 1996, the American Pain Society advocated that healthcare providers should screen for pain as “fifth vital sign” (P5VS initiative), which was subsequently adopted by the US Veterans Health Administration in a national strategy to include mandatory pain screening and pain-related patient satisfaction questions (25). Coinciding with the P5V5 initiative, Purdue Pharma brought to market OxyContin, which it was advertising as a novel opioid pain medication that was long-acting and therefore “less prone to abuse” (26, 27).

As early as 2003, the US Drug Enforcement Administration established that Purdue Pharma’s “aggressive, excessive and inappropriate” product marketing under-communicated risks and “very much exacerbated” abuse of OxyContin (23). OxyContin marketing sought to normalize prescription opioid use by shaping public opinion through direct consumer-advertising with the promise of pain-free living (such as the slogan “There Can Be Life with Relief”) (28). In parallel, thousands of prescribers were targeted with misinformation, including a 2007 American Medical Association training on pain management “made possible by an educational grant from Purdue Pharma” (29, 30).

At all-expenses-paid seminars involving supposedly independent “key opinion leaders” such as pain management specialists and patient charities, doctors were encouraged to “overcome their opiophobia” and prescribe opioids for a wider range of medical indications (31). As Pettigrew et al. (30) write: “Purdue Pharma set up “pain groups” as part of a wider “pain movement” to promote the use of the opioid OxyContin to treat a wide range of conditions – from cancer and severe pain management initially to more minor conditions, and increasingly higher doses – while denying it was addictive.” In Australia for example, Mundipharma sponsored over 3,000 pain-related educational events from 2011 to 2015 which targeted doctors and nurses in the interest of opioid promotion (32). An internal Purdue Pharma analysis found that physicians who attended these events wrote more than double the number of OxyContin prescriptions of non-attendees (23). The role of pharmaceutical industry in provider education is thus problematic (31).

#### 1.1.3 Diversionary tactics

By using self-serving slogans such as “drink responsibly” or “smoke responsibly,” industry tend to shift responsibility onto the individual to detract from corporate harm. Notably, “responsible” behavior is left purposely vague in these slogans (5).

In its response to the opioid crisis, Purdue and its owners, the Sackler family, strategically blamed consumers for their “irresponsible use” of OxyContin. As Radden Keefe (33) writes in his history of the opioid epidemic: “People did abuse these drugs, Arthur [Sackler] conceded. But the real explanation for this phenomenon was not any intrinsically addictive properties of the drugs themselves. Rather it was a reflection of the addictive personalities of the users themselves. What Purdue should do, he decreed, was “hammer on the abusers in every way possible.” They are “the culprits” he declared. “They are reckless criminals.””

On 30 May 2023, a New York court of appeals granted immunity to the Sackler family, ruling in a $6 billion USD settlement agreement (34) that all family members will be protected from current and future lawsuits over their role in Purdue Pharma’s opioid business (35). The case has been moved to the US Supreme Court where it is currently on hold and will be reviewed in December 2023 (36).

### 1.2 Policy inertia: what are the reasons for the slow response to man-made crises?

Another commonality between the North American opioid epidemic and other man-made crises is the collective experience that “had we intervened sooner the current situation could have been different or perhaps even averted” (7). What are then the reasons for our historically slow response to silent epidemics that unfold before our eyes? And how does our response to man-made crises differ from our management of “natural epidemics”?

A particular challenge to public health arises from the fact that, at the level of individual trajectories of drug use, adverse health effects can often only be detected many years after first drug exposure. Rhodes and Lancaster (37) make a compelling case that the short-term focus of early warning systems and outbreak detection is unsuitable for the description of the “slow death” or “slow emergency” of opioid overdose. In Europe, the average age of drug-induced deaths is 41 years (38), occurring likely more than two decades after the onset of drug use in many individuals. Statistically speaking, overdose death is a rare event (39) relative to the number of people who use opioids and their frequency of use, which makes time-sensitive changes in the rate of overdoses difficult to detect at population level.

The duration of the time window between initial use of a drug and the occurrence of drug-related harms will likely also depend on the drug’s potency and its abuse potential. For instance, the arrival of illicitly manufactured fentanyl (a highly potent full opioid agonist) on the drug market in US cities around the year 2013 almost immediately led to a rise in overdose fatalities, which is now considered the onset of the “third wave” of the US opioid epidemic (40). Tramadol, by contrast, is considered a ‘weak opioid’ with mixed mechanism and lower abuse potential than other opioid analgesics (41, 42). According to the World Drug Report (9) an epidemic of non-medical use of tramadol is currently unfolding along trade routes in North Africa, West Africa, the Near and Middle East and South-West Asia, posing great health risks. First indicators of harm have included the increase in treatment demand for tramadol use disorder in some African as well as local reports of high rates of tramadol involvement in traffic accidents (9, 43). However, due to lack of epidemiological data on drug use and routine toxicology testing in the affected regions, the current scale of this epidemic in the making is unknown, and public health interventions remain largely absent.

In man-made crises, availability of epidemiological data is not enough for change to occur. As early as 2003, the US FDA issued a warning letter to Purdue Pharma over its omission and minimization of the safety risks associated with OxyContin in the product’s marketing materials (28). Two years later, Cicero et al. (44), reported an increase in nonmedical use of OxyContin “among street and recreational drug users” based on epidemiological surveillance data (RADARS; 2002–04), concluding that “steps need to be taken to reduce prescription drug abuse.”

Yet, policy inertia from both North American regulatory agencies (Health Canada, US FDA) and relevant public health bodies prevailed. This enabled Purdue Pharma’s ongoing OxyContin sales to cause addiction and death in the population – at the expense of individuals, governments, and non-governmental organizations having to meet these costs.

Using the notion of inductive risk to illustrate the moral severity of errors at the post-marketing approval stage, Bavli and Steele (45) argue that Health Canada could have prevented significant public health harm if it had applied a less strict standard of evidence as requirement for revisions to the OxyContin product monograph, which understated the risk profile of the drug.

Within the addiction care system, many evidence-based practices were available in the first wave of the opioid epidemic, including the expansion of access to medications for opioid use disorder (methadone or buprenorphine treatment; naloxone for overdose reversal) and harm reduction measures (e.g., needle and syringe programs). Still, these were not systematically being implemented until *after* the Unites States entered the second and third wave of the opioid epidemic, with increases in heroin- and fentanyl-related deaths beginning in 2010 and 2013, respectively (46). In the meantime, opioid overdoses and mortality had escalated, leaving North American healthcare systems increasingly unable to cope.

### 1.3 What is the role of industry in science?

For pharmaceutical industry, scientific publication of results from clinical trials is a key marketing strategy for drugs. However, in clinical trials conducted or sponsored by industry, potential bias in the selection of study design and outcomes as well as lack of transparency in reporting threaten patient safety. As Bero (47) writes, “empirical research demonstrates that pharmaceutical […] industry funding biases human studies towards outcomes that are favorable to the sponsor, even when controlling for other biases in the methods.”

Indeed, in their retrospective analysis of regulatory data submitted by Purdue Pharma to Health Canada for the approval of OxyContin, Pappin et al. (48) determined that “[n]one of the trials sponsored by Purdue Pharma sought to meaningfully assess the risks of misuse or addiction associated with OxyContin. The trials were short in duration (maximum length was 24 days) and only assessed safety and efficacy of a 12-h dosing interval. Also, the two trial reports that explicitly mentioned (but did not formally evaluate) the risk of misuse were not published.”

Importantly, Purdue Pharma external funding awards were not limited to specific project grants (e.g., for clinical trials) but has also included institutional donations from Sackler family charities to leading universities in the United Kingdom and US (49). A recent New York Times (2023) investigation documented that – In a clear conflict of interests – the National Academies of Sciences, Engineering and Medicine, a nongovernmental institution, received approximately $19 million in Sackler donations while advising the US federal government on opioid policy (50).

These institutional donations are akin to former tobacco industry funding of universities to conduct studies into various health topics – so-called “red herring” research that could serve as distraction from the corporate agenda of boosting tobacco sales (30, p. 53). Within the opioid industry, corporate funding of service user initiatives (51) or the recent Purdue Pharma announcement of investment into ‘opioid rescue medicines’ (35) arguably fall in the same category. Analogous to pharmaceuticals and tobacco, the negative impact of the “funding effect” on research agendas and integrity has been documented for the alcohol and, more recently, cannabis industries (52).

## 2 Discussion and conclusion

The origins of the prescription opioid epidemic may be traced to innovations in drug development with the promise of improved pain management. However, through multiple factors, including fraudulent marketing from pharmaceutical industry as well as policy inertia, the resulting crisis represents a multi-system failure of regulation exploited by corporate greed.

Unlike natural disasters or viral disease outbreaks that are confined in space or time, manmade crises develop gradually as commercial determinants affect health through proximal and distal pathways. Due to a lag in outcomes, their onset and initial evidence of harm are more challenging to identify, which can hinder early public health response.

The prolonged US opioid crisis has accounted for a death toll far higher than the country’s natural disasters. Still, it was only declared a Public Health Emergency in 2017 (53) – more than two decades after OxyContin became commercially available. In the meantime, opioid prescribing and related deaths continued to disproportionately affect US communities with greater levels of deprivation (54). As we have demonstrated through application of the CDOH framework, the prescription opioid epidemic exemplifies what West and Marteau (4) described as “the tension between wealth- and health-creation,” where public health is fundamentally at odds with the profit motive of industries producing addictive commodities.

To contend with industry influence in science, including the growing role of industry-academia collaborations, society needs to set up more stringent mechanisms for declaring conflicts of interests at individual and institutional level as well as corporate lobbying activities. This becomes increasingly relevant for the addictions field as corporations are heavily investing in lobbying for exemptions from the international prohibition of cannabis and psychedelics, promoting their therapeutic or recreational use (55, 56). Moreover, real-world data as well as transparency in the public documentation of clinical trial data (57) and regulators’ data interpretation are needed (48).

To prevent future crises involving addictive commodities from occurring, regulatory agencies will require appropriate staffing capacity to review safety data and promotional materials (31). At the post-approval stage, regulators should apply a less strict standard of evidence of product-related harm in individuals to avoid population-level adverse consequences (45). If a crisis develops, industry-independent funding for rapid assessments will be crucial to gather evidence and inform health policy.

Finally, at the benefit of corporate profits, medical systems are designed to initiate patients on drugs, not take them off (58). Despite minimal evidence of their long-term effectiveness, overprescribing of opioids has put patients at risk of dependence, side effects, and drug–drug interactions (59). Research funding is urgently needed to study safe strategies for deprescribing opioids and other medications with potential for addiction or physical dependence.

To quote the conceptualization of the CDOH by Gilmore et al. (5), pharmaceutical companies will “need to meet the true costs of the harm they cause, governments will need to exercise their power in holding [these] commercial entities to account, and [clinical] norms [and practices] need to be reshaped in the public interest.”

## Data availability statement

The original contributions presented in the study are included in the article/supplementary material, further inquiries can be directed to the corresponding author.

## Author contributions

RM: conceptualization, writing of original draft, review and editing. DE: conceptualization and review. SS: conceptualization and review. TC: conceptualization, review and editing, and supervision.
